# Cervical sympathetic trunk transection alleviates acute lung injury caused by intestinal obstruction via inhibition of phospholipase A_2_ in rats

**DOI:** 10.1186/s12871-022-01814-2

**Published:** 2022-08-23

**Authors:** Zhengfeng Gu, Lian Xin, Huizhi Yu, Shunmei Lu, Jinbo Wu, Hui Wang, Dongxiao Huang, Chunxiao Hu

**Affiliations:** Department of Analgesia and Anesthesiology, Nanjing Medical University Affiliated Wuxi People’s Hospital, Wuxi, China

**Keywords:** Acute lung injury, Intestinal obstruction, Phospholipase A_2_, Stellate ganglion block

## Abstract

**Background:**

Intestinal obstruction can result in inflammatory injury to distant organs, especially the lungs. Stellate ganglion block (SGB) provides sympathetic nervous homeostasis and inhibits the systemic inflammatory response. This study aimed to investigate whether SGB can alleviate acute lung injury by inhibiting phospholipase A_2_ expression in rats.

**Methods:**

Thirty healthy male Sprague–Dawley rats were divided into three groups: C group (sham-operated); CLP group (cecal ligation and puncture with intestinal obstruction), and cervical sympathetic trunk transection (CSTT) group (transection of the cervical sympathetic trunk following CLP).Arterial blood samples were obtained to determine the ratio of partial arterial pressure of oxygen (PaO_2_) to fraction of oxygen in inspired air (FiO_2_). Venous blood samples were used to evaluate the serum concentrations of chemokines, tumor necrosis factor (TNF)-α, interleukin (IL)-6, and IL-10 using enzyme-linked immunosorbent assays. Following euthanasia, the lungs were isolated to estimate the wet/dry lung weight (W/D) ratio, evaluate the pathological damage to lung tissues on microscopy, and determine secretory-type phospholipase A_2_ (sPLA_2_) expression using western blotting.

**Results:**

Rats in the CLP group showed increased fatigue, decreased activity levels, and coarse, gray hair. The levels of chemokines, TNF-α, and IL-6 in the CLP and CSTT groups were higher than those in the C group. However, the levels were lower in the CSTT group than those in the CLP group. IL-10 levels in the CLP group were higher and lower than those in the C and CSTT groups, respectively. W/D ratios and PaO_2_/FiO_2_ in the CLP and CSTT groups were higher than those in the C group, whereas these ratios in the CSTT group were lower than those in the CLP group. No lung injury was noted in group C, and the lung injury scores were lower in the CSTT group than those in the CLP group. sPLA_2_ expression levels in the CLP group were higher than those in the C group, whereas these levels in the CSTT group were lower than those in the CLP group.

**Conclusions:**

sPLA_2_ overexpression in the lungs may be a pathogenic factor in acute lung injury. CSTT alleviated acute lung injury by inhibiting sPLA_2_ expression.

**Supplementary Information:**

The online version contains supplementary material available at 10.1186/s12871-022-01814-2.

## Background

Secretory-type phospholipase A_2_ (sPLA_2_) and its products play critical roles in inflammatory diseases, such as acute lung injury (ALI). Several isoforms of _S_PLA_2_ are upregulated in multiple ALI models, and some isoforms play unique roles in the regulation of the pathophysiology of ALI [1]. Intestinal obstruction may result in bacterial translocation and the production of inflammatory factors, which may result in ALI. Stellate ganglion block (SGB) can regulate the sympathetic nervous system and inflammatory response [2, 3]. Therefore, in this study, we aimed to investigate whether cervical sympathetic trunk transaction (CSTT) can regulate inflammation via inhibition of sPLA_2_ secretion.

## Methods

### Ethics statement

All animal experiments were approved by the Ethics Committee of the Wuxi People’s Hospital (approval number: MS201916).

### Animals

Thirty healthy male Sprague–Dawley rats (270–320 g) were acquired from Changzhou Cavens Laboratory Animal Co., Ltd. (Changzhou, Jiangsu, China) and housed in specific pathogen-free conditions with animal service from the Laboratory Center of Jiangsu Lung Transplantation in Wuxi. The room temperature was set at 23–25 °C with a 12-h day/night cycle, and the rats (*n* = 5 per cage) were acclimatized for 7 days with ad libitum access to water and food before the experiments [4]. The rats were fed and cared for by a full-time technician, who coded the cages and rats. The rats were randomly assigned to three groups using a computer-based random order generator (*n* = 10 each): (1) C group, in which the rats underwent abdominal and cervical incisions and sutures; (2) cecal ligation and puncture (CLP) group, in which the rats underwent CLP; and (3) CSTT group, in which the rats underwent isolation and transection of the sympathetic nerve trunk following CLP. For each animal, five different investigators were involved and had the following roles.The first investigator performed the procedures including sample collection based on the randomization table. This investigator was the only person aware of the treatment group allocation. The second investigator was responsible for performing blood gas analysis and serum biochemical assay, and the third investigator performed the western blot analysis and histopathological test. Finally, the fourth investigator (also unaware of treatment) assessed the animals’ behavior, and the fifth investigator performed data analysis.

### Surgical procedures

The rats were anesthetized using 5% sevoflurane on an anesthesia machine for small animals followed by intraperitoneal injection of pentobarbital sodium (30 mg/kg) [5, 6]. All the rats in this trial maintained autonomous respiration and air inhalation during the whole procedure.They were maintained in a supine position on a heating table mat (AUX Group, Ningbo, Zhejiang, China) with the temperature set to 37 °C. Hair over the abdomen and around the neck was shaved using an electric haircutter. Anesthesia was verified by testing limb immobility via forceps clamping of the skin. The skin over the abdomen and neck was sterilized using 75% alcohol. Ophthalmic scissors were used for the incisions of the skin, abdominal muscles, and peritoneum along Hunter’s line of the lower abdomen at a width of 1.5–2.0 cm. An incision along Hunter’s line can prevent vascular injury and bleeding. The cecum, usually located in the right upper abdomen, was extracted using anatomical forceps. A 3–0 silk suture was passed through the mesocecum, and the cecum was ligated at its upper third adjacent to its root [7]. The cecum was then bilaterally punctured by inserting an 18-gauge needle through the cecal wall followed by cecal pressing with sterile cotton swabs and extrusion of the intestinal contents through the punctured openings. The cecum was returned to the abdominal cavity, and the peritoneal and skin incisions were sutured layer-by-layer using 3–0 absorbable sutures. Sham-operated mice in the C group underwent identical procedures except for CLP. All animals received an abdominal infusion of normal saline (1 mL) after closure of the abdominal wall.

The mice in the CSTT group underwent identical procedures for CLP with additional CSTT [8, 9]. The ventral neck was incised using ophthalmic microscissors to expose the platysma myoides and left sternocleidomastoid. The triangle of the left sternocleidomastoid was bluntly dissected using the ophthalmic microscissors until the carotid artery sheath was exposed. The carotid artery was isolated from the nerve trunk using ophthalmic microforceps. The top end of the forceps was arc-shaped with a diameter of 0.3 mm. With the vagus nerves isolated from the sympathetic nerve trunk using the ophthalmic microforceps, the sympathetic nerve trunk was transected using ophthalmic microscissors. The platysma myoides and skin were closed using 3–0 absorbable sutures. The index of a successful model establishment was the development of Horner’s syndrome, with features such as blepharoptosis, narrowing of the palpebral fissure, miosis, and canthus secretions. Conversely, the absence of Horner’s syndrome was considered an indication of a failed model, and another experiment was warranted. If the rats died during the procedure, another experiment was conducted until the desired number was achieved.

The rats were returned to their cages followed by care and ad libitum access to food and water in the laboratory for 24 h. The animals’ behavior; wet/dry lung weight ratio (W/D); oxygenation index (expressed as the ratio of arterial partial pressure of oxygen [PaO_2_] to oxygen concentration [FiO_2_]); lung injury scores assessed by histopathology analysis; serum concentrations of chemokines, tumor necrosis factor (TNF)-α, interleukin (IL)-6, and IL-10; and sPLA_2_ levels were assessed.

### Blood gas analysis

After 24 h, the rats were anesthetized, and the lower abdomen was opened through the original incision. The small intestine was extracted, and the mesentery was bluntly and gently isolated using sterilized cotton swabs. The abdominal aorta and inferior vena cava were then exposed. Blood from the aorta was obtained using a 1-mL syringe for arterial blood gas analysis. The oxygenation index was evaluated by dividing PaO_2_ by FiO_2_. We regarded FiO_2_ in the air as 21%. Venous blood was obtained from the inferior vena cava and stored in an anticoagulant-coated tube. Venous blood was centrifuged, and the serum was stored in a refrigerator for subsequent analysis.

### Histopathological analysis

The rats were euthanized by cervical dislocation. The pulmonary artery was irrigated with normal saline until the absence of blood was noted from the lungs. With the superior lobe of the right lung isolated, water and blood were removed using a filter paper, and the wet weight was measured. Then, the sample was kept in a drying oven at 120 °C for 24 h; subsequently, the dry weight was estimated, and the W/D was calculated. The middle lobe of the right lung was dissected, fixed with 10% formaldehyde solution, embedded in paraffin, microtomed into slices, and stained with hematoxylin–eosin. The slides were observed under a light microscope (100 × and 200 × magnifications). Histological changes were evaluated by a pathologist blinded to the experimental conditions. The degree of lung injury was graded using a histological scoring system [10]. Edema, alveolar and interstitial inflammation and hemorrhage, atelectasis, necrosis, and hyaline membrane formation were scored on a 5-point scale as follows: 0, no injury; 1, injury in 25% of the viewing field; 2, injury in 50% of the viewing field; 3, injury in 75% of the viewing field; and 4, injury throughout the field. Three different viewing fields per slide were analyzed, and the mean score of the three viewing fields was considered the score of the slide. The final lung injury score was obtained by summing these scores [11].

### Serum biochemical indices

The serum concentrations of chemokines, TNF-α, IL-6, and IL-10 were determined using an enzyme-linked immunosorbent assay (ELISA) kit (Neobioscience, Nanjing Proteinbio Technology Co., Ltd., China) according to the manufacturer’s instructions [12]. The plates were read using a microplate reader at a wavelength of 450 nm [13].

### Western blotting

sPLA_2_ levels were determined using western blotting according to the instructions of the Protein Assay Kit (Abcam, Cambridge, Univ Biotechnology Co., Ltd., Shanghai, China, ab139692) [14]. The left lung was dissected; 100 mg was cut into pieces before being placed into the centrifuge tube, and 1 mL of lysis solution was injected into the tube. A total of 50 μL of lysis solution included 40 μL of radioimmunoprecipitation assay, 5 μL of the protease inhibitor, 5 μL of the phosphatase inhibitor, and 0.5 μL of phenylmethanesulfonyl fluoride. Two 50-mL beakers were prepared. One beaker was filled with normal saline as a wash homogenizer, and another one was filled with ice to keep the centrifuge tube filled with lung tissue cool. Homogenization was performed for 2 s. The centrifuge was placed in a refrigerator at 4 °C for more than an hour before being used. The lysed tissue was centrifuged for 10 min at 15,000 rpm and 4 °C after being lysed for 30 min at 4 °C. About 800 μL of supernatants were obtained.Samples from the cell supernatants were subjected to 15% lauryl sodium sulfate–polyacrylamide gel electrophoresis followed by immunoblotting onto a polyvinylidene difluoride membrane. Nonspecific binding sites were blocked via incubation with 5% skim milk in Tris-buffered saline containing 0.1% Tween-20 (TBS-T) for 4 h at 4 °C. The membranes were then incubated overnight. After rinsing with TBS-T, the blots were incubated for 2 h at room temperature. The immunoreactive bands were visualized using an enhanced chemiluminescence detection kit, and the intensities were analyzed using an image processing program. Equal protein loading was confirmed using horseradish peroxidase-conjugated anti-mouse IgG (ab205719; Abcam, Cambridge, UK) as the secondary antibody.

### Statistical analysis

Values are expressed as mean ± standard deviation of three experiments. Comparisons were assessed using repeated measurement variables (within-subjects factors) followed by an independent samples *t*-test. Data were tested using the D’Agostino–Pearson test for normal distribution. Data were analyzed using MedCalc (version 20.110–32-bit;MedCalc Software Ltd., Ostend, Belgium). The level of statistical significance was set at *p* < 0.05.

## Results

### Behavioral alterations

The rats in the CLP group showed increased fatigue, reduced activity levels, and coarse, gray hair compared to those in the C and CSTT groups. The rats in the CSTT group demonstrated narrowing of the palpebral fissure in one eye and secreta at the canthus, which confirmed the successful establishment of the model.

### Histopathological changes

**Fig. 1:**
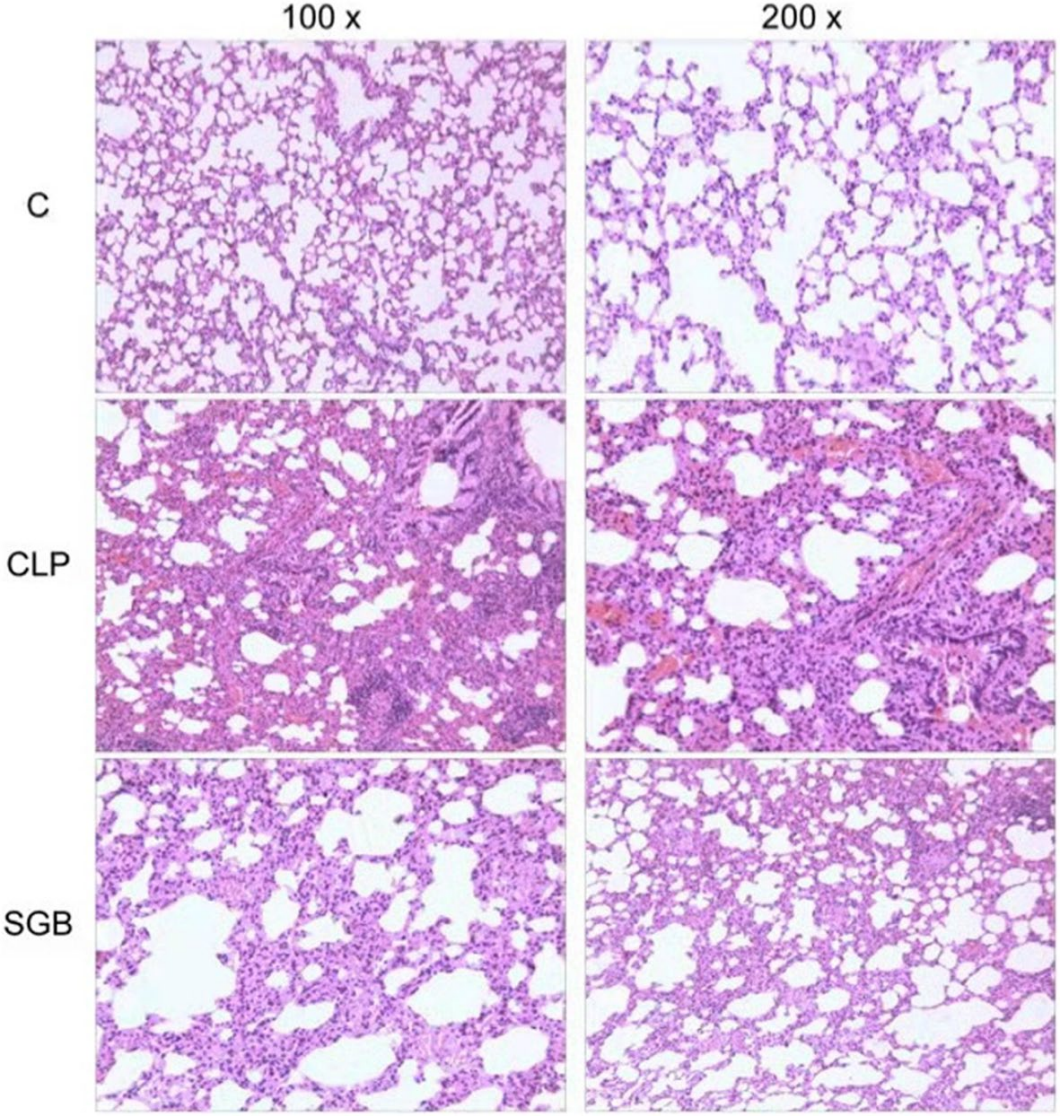
Histopathological images of lung tissue samples from each experimental group (100 × and 200 × magnifications, hematoxylin & eosin staining)

We evaluated the profiles of the lung injuries caused by CLP. Lung tissues from the C group demonstrated normal structures without histological changes under a light microscope (100 × and 200 × magnifications; Fig. 1). The lung tissues in the CLP group showed severe histological lesions, including reduced alveolar cavities, increased area of the pulmonary interstitial space due to edema, interstitial hyperemia, hemorrhage, and evident infiltration of inflammatory cells, which demonstrated the successful establishment of the CLP-induced ALI model. However, the lung tissues in the CSTT group demonstrated mitigated interstitial edema, reduced pulmonary interstitial space, and decreased infiltration of inflammatory cells compared to those in the CLP group (Fig. 1). There were significant differences in the pathological scores between the CLP and CSTT groups (*p* < 0.001; Table 1).

**Table 1:** Wet/dry lung weight ratios (W/D), PaO_2_/FiO_2_ ratios, lung injury scores, and sPLA_2_ levels (*n* = 10)

**Group**	**W/D**	**PaO**_**2**_**/FiO**_**2**_	**Lung injury scores**	**sPLA**_**2**_** (ng/L)**
C	4.09 ± 0.19	275.5 ± 11.7	0	1.08 ± 0.01
CLP	5.11 ± 0.15 ^a^	213.4 ± 22.1 ^a^	7.01 ± 0.41	1.68 ± 0.04 ^a^
CSTT	4.73 ± 0.22 ^ab^	264.5 ± 22.3 ^b^	4.53 ± 0.23 ^b^	1.46 ± 0.02 ^ab^

Values represent mean ± standard deviation (*n* = 10)

*PaO*_2_ Partial arterial pressure of oxygen, *FiO*_2_ Fraction of oxygen in inspired air, *sPLA*_2_ Secretory-type phospholipase A_2_, *C* Control, *CLP* Cecal ligation and puncture, *CSTT* Cervical sympathetic trunk transection

^a^ *p* < 0.001 versus C group

^b^ *p* < 0.001 versus CLP group

### W/D and PaO2/FiO2

The W/D and PaO_2_/FiO_2_ ratios in the CLP and CSTT groups were greater than those in the C group, whereas the ratios in the CSTT group were lower than those in the CLP group (*p* < 0.001; Table 1).

### Chemokines, TNF-α, IL-6, and IL-10

We determined the levels of inflammatory factors to assess the inflammatory reactions. The expression levels of chemokines, TNF-α, and IL-6 in the CLP and CSTT groups were higher than those in the C group (*p* < 0.001; Table 2), whereas these cytokine levels in the CSTT group were lower than those in the CLP group (*p* < 0.001; Table 2). IL-10 levels in the CLP group were higher than those in the C group. Furthermore, IL-10 levels in the CSTT group were higher than those in the CLP group (*p* < 0.001; Table 2).

**Table 2:** Serum concentrations of CK, TNF-α, IL-6, and IL-10 (*n* = 10)

**Group**	**CK (ng/L)**	**TNF-α (ng/L)**	**IL-6 (ng/L)**	**IL-10 (ng/L)**
C	23 ± 11	60 ± 12	66 ± 11	61 ± 13
CLP	95 ± 14 ^a^	223 ± 22 ^a^	198 ± 14 ^a^	99 ± 12 ^a^
CSTT	69 ± 12 ^ab^	152 ± 15 ^ab^	149 ± 12 ^ab^	139 ± 16 ^ab^

Values are presented as mean ± standard deviation (*n* = 10)

*C* Control, *CLP* Cecal ligation and puncture, *CSTT* Cervical sympathetic trunk transection, *CK* Chemokines, *TNF-α* Tumor necrosis factor α, *IL-6* Interleukin-6, *IL-10* Interleukin-10

^a^ *p* < 0.001 versus C group

^b^ *p* < 0.001 versus CLP group

### Western blotting of sPLA2

**Fig. 2:**
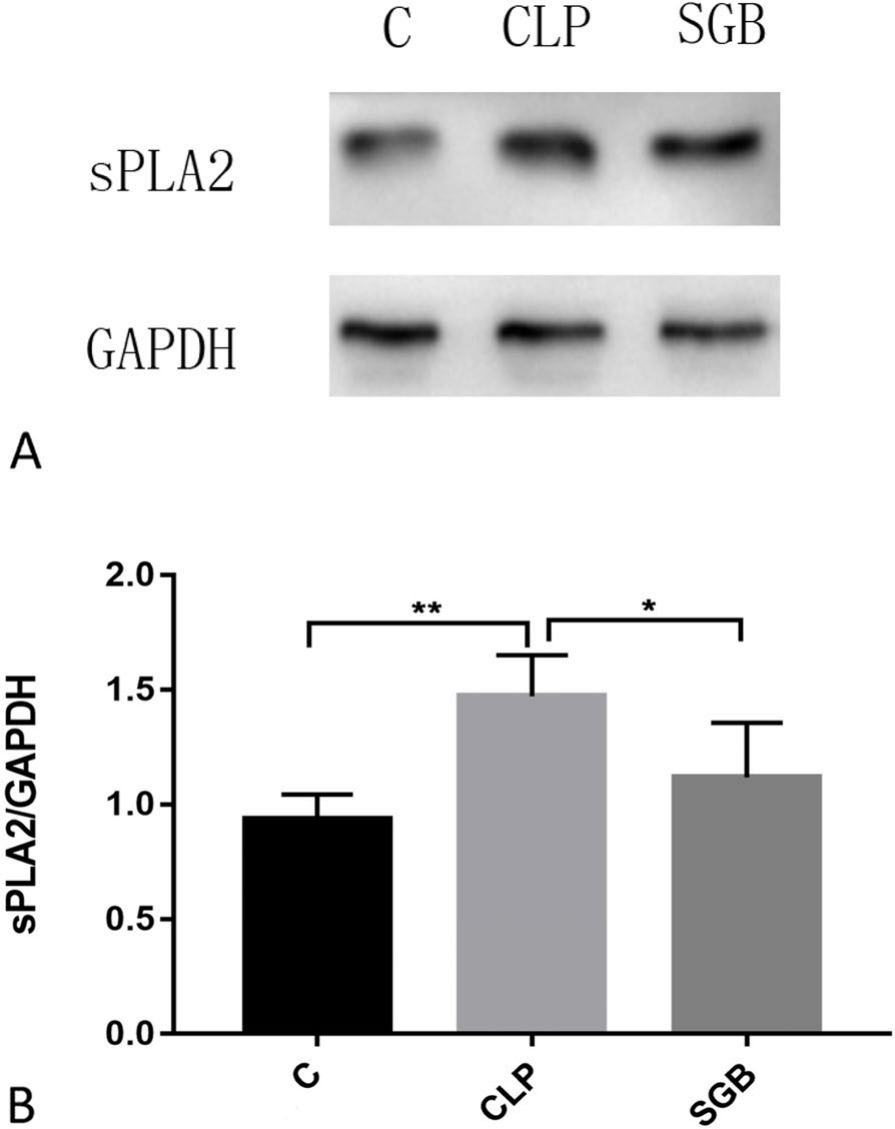
Protein levels of secretory-type phospholipase A_2_ (sPLA_2_) determined using western blotting. **A** Representative sPLA_2_ bands (full-length blots are presented in Additional file 1). **B** Concentrations of sPLA_2_normalised to GAPDH expression. ^*^ and ^**^p < 0.001

Western blotting was performed to investigate whether CSTT affected the expression of sPLA_2_. The protein levels of sPLA_2_ in the lung tissues of the CLP group were higher than those in the C group, whereas the sPLA_2_ levels in the CSTT group were lower than those in the CLP group (*p* < 0.001; Fig. 2A and B, Table 1).

## Discussion

ALI is one of the commonest complications of intestinal obstruction and is associated with high morbidity and mortality [6]. Intestinal obstruction is a common disease of the digestive tract, particularly in children and elderly patients. The release of gut-derived detrimental factors into the circulation has been implicated in the development of ALI [5]. The ALI model induced using CLP in laboratory animals can perfectly imitate the clinical manifestations of intestinal obstruction, peritonitis, and ALI [5, 6]. The CLP-induced animal model is acknowledged as the standard model of sepsis, which justifies our employment of CLP in the establishment of a model of ALI.

Some pharmaceutical agents, such as ulinastatin and cucurbitacin, have been reported to have positive effects on the prevention and treatment of ALI. In recent years, SGB has been widely adopted in clinical therapeutics for various diseases for its inhibitory effects on inflammatory reactions caused by trauma, infection, shock, and major surgery [3, 15, 16]. CSTT also improves pulmonary function by regulating the homeostasis of the autonomic nervous system [17]. Our study identified that CSTT improved pulmonary oxygenation (improved PaO_2_/FiO_2_) and mitigated lung edema by decreasing pulmonary permeability and exudation (lower W/D ratio and lung injury scores). Furthermore, SGB effectively inhibited the inflammatory factors by downregulating chemokines, TNF-α, and IL-6 levels, while upregulating the expression of IL-10, which inhibits the release of inflammatory factors, such as TNF-α, IL-1β, IL-6, and IL-8. IL-10 is a kind of anti-inflammatory mediator. Infection activates the sympathetic nervous system, and the latter downregulates or inhibits the expression of IL-10. CSTT relieves the inhibition of IL-10 expression by the sympathetic nervous system [18]. These results are supported by the findings of previous studies [3, 17]. Increased activity of the rats in CSTT group demonstrated the attenuation of inflammatory reactions.

The oxygenation index, calculated as PaO_2_/FiO_2_, is an important indicator of lung function. Lower values of PaO_2_/FiO_2_ in the CLP group compared with those in the C group confirm the CLP-induced impairment of lung oxygenation. Despite the decreased PaO_2_/FiO_2_ in the CSTT group (versus the CLP group), CSTT increased the oxygenation of the injured lung. Our pulmonary histopathological measurements revealed that CSTT alleviated alveolar epithelial edema, decreased the percolate in the alveolar cavity, and reversed the narrowing of alveolar walls. These findings suggest that CSTT can protect lung function by reducing the levels of cytokines, such as IL-6 and IL-8. Our results are consistent with prior findings by Chen et al. [3].

To date, more than 30 enzymes that possess PLA_2_ or related activities have been identified. Approximately one-third of these enzymes belong to the sPLA_2_ family, which comprises low molecular weight, Ca^2+^-requiring, secreted enzymes with a histidine/aspartate catalytic dyad and serve specialized biological roles [19]. To date, eleven sPLA_2_ isoforms (IB, IIA, IIC, IID, IIE, IIF, III, V, X, XIIA, and XIIB) have been identified in mammals and are subdivided into three major categories: one containing the conventional sPLA_2_ enzymes (I/II/V/X) and two groups of atypical sPLA_2_ enzymes (III and XII). Phospholipase A (PLA) comprises a supergroup of esterase enzymes present in all human cells and plays a key role in mediating the production of free fatty acids and lysophospholipids from glycerophospholipids. The isoforms of PLA fall into six main groups: cytosolic PLA, calcium-independent PLA, secretory PLA, lysosomal PLA, adipose-specific PLA, and platelet-activating factor acetylhydrolase [20]. The molecular structure of secretory PLA consists of 6–8 disulfide bonds and shares a histidine/aspartate active site with a calcium cofactor for catalysis. Secretory PLA has been demonstrated to have a wide variety of functions in the body. It possesses potent antiviral properties, as well as antibacterial activities against a wide spectrum of Gram-positive and Gram-negative bacteria. The mechanism involves the penetration of the peptidoglycan cell wall by the degradation of membrane phospholipids. The antiviral mechanism of secretory PLA involves inhibition of chemokine receptors, which ultimately prevents viral entry into host cells. ‘Sepsis’ is currently identified as ‘a life-threatening condition that arises when the body’s response to an infection injures its own tissues and organs’ [21]. Initiated by an invading pathogen, generally represented by bacteria and, less frequently, by viruses or fungi, sepsis results in an inflammatory process in which the body’s own response has a deleterious effect on itself [22]. Pathogen infection is the condition of ‘sepsis’ and activates sPLA_2_. Secretory PLA_2_causes then the release of inflammatory factors. The tissue is damaged when the concentration of inflammatory factors in the body reaches a certain level. Secretory PLA_2_ is favorable for resisting viruses and bacteria, but it damages the normal cell membrane. Thus, attenuating the inflammatory process bydecreasing sPLA_2_ expression protects the body. Although inflammation and tissue damage are hallmarks of sepsis, the specific mechanisms linking inflammation, injury, and outcomes remain unclear [23]. Our findings demonstrated that CSTT could inhibit the concentration of serum chemokines and, consequently, alleviate ALI via inhibition of chemokines. Nevertheless, the mechanism of inhibition of chemokine production by CSTT remains unclear and requires further research. Phospholipase A_2_hydrolyzes phospholipids and initiates the production of inflammatory lipid mediators [24]. Secretory PLA_2_ contributes to pulmonary diseases [25, 26]. Moreover, sPLA_2_-V and sPLA_2_-X can potently hydrolyze phosphatidylcholine in vitro,which highlights that the mechanism of airway injury, at least partially, includes hydrolytic degradation of lung surfactants. Owing to the well-known significance of secretory PLA in the pathogenesis of several inflammatory diseases, attempts to synthesize inhibitors of the enzyme are underway for quite some time for the treatment of some of these diseases. Phosphodiesterase inhibitors exert a broad spectrum of favorable effects that are potentially beneficial in ALI [27]. Phospholipase A_2_ inhibitors are beneficial in the treatment of ALI associated with bacterial infections [28, 29], thus implying that inhibition of phospholipase A_2_ will produce advantageous results in ALI. Our findings demonstrated that CSTT may decrease sPLA_2_ concentrations in lung tissues. PLA_2_ acts as an acute-phase protein, and its serum concentration is related to the mortality intoxic shock and multiple organ failure, thus rendering it important in establishing the diagnosis and estimating the prognosis [30].

There are some limitations to this study. First, although this study demonstrated that CSTT inhibits sPLA_2_ expression, further studies are needed to clarify the mechanism underlying this observation. Second, this study was conducted in an animal model. Third, we did not evaluate the heart rate, blood pressure, and oxygen saturation. Therefore, our resultsshould be clinically confirmed by SGB.

## Conclusions

Due to the potent anti-inflammatory activity of CSTT, its supplementation can effectively improve pulmonary oxygenation, mitigate pulmonary edema, and inhibit inflammatory infiltration by reducing the production of sPLA_2_. These results reflect the beneficial effects of SGB in the clinical treatment of ALI.

## Supplementary Information

**Additional file 1. **Western blot images.

## Data Availability

All data generated or analyzed during this study are included in this published article and are available from the corresponding author on reasonable request.

## Abbreviations

ALI: Acute lung injury
CLP: Cecal ligation and puncture
CSTT: Cervical sympathetic trunk transection
ELISA: Enzyme-linked immunosorbent assay
IL: Interleukin
PaO2/FiO2: Arterial partial pressure of oxygen to oxygen concentration ratio
PLA: Phospholipase A
SGB: Stellate ganglion block
sPLA2: Secretory-type phospholipase A2
TBS-T: Tris-buffered saline containing 0.1% Tween-20
TNF-α: Tumor necrosis factor-α
W/D: Wet/dry weight ratio
