# Supplementary material for: Cervical sympathetic trunk transection alleviates acute lung injury caused by intestinal obstruction via inhibition of phospholipase A2 in rats
Source: BMC Anesthesiol. 2022 Aug 23;22:270. doi: 10.1186/s12871-022-01814-2 (PMC9400334; doi:10.1186/s12871-022-01814-2)

**Western Blot Images**

SPLA2： C1 CLP1 SGB1 SPLA2：C2 CLP2 SGB2


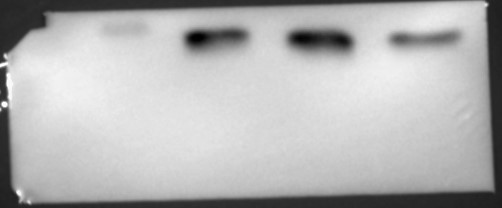

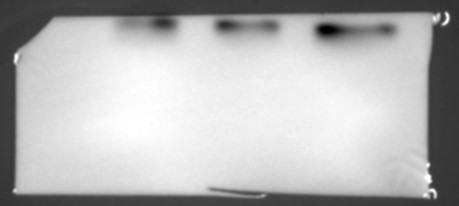


SPLA2： C3 CLP3 SGB3 SPLA2：C4 CLP4 SGB4


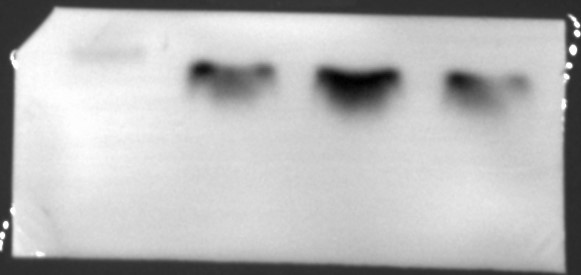

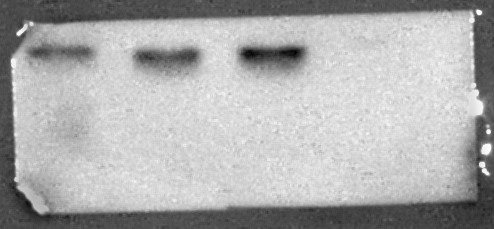


SPLA2： C5 CLP5 SGB5


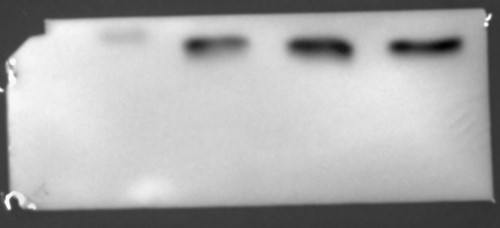


SPLA2： C6 CLP6 SGB6 SPLA2：C7 CLP7 SGB7


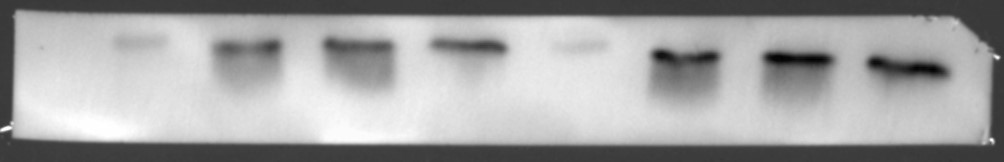


SPLA2： C8 CLP8 SGB8 SPLA2：C8 CLP8 SGB8


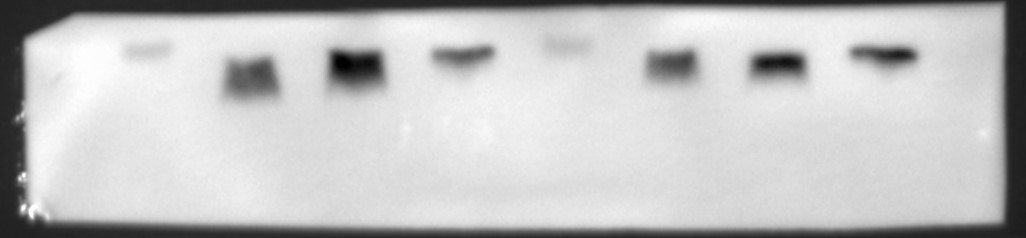


SPLA2： C9 CLP9 SGB9 SPLA2：C10 CLP10 SGB10


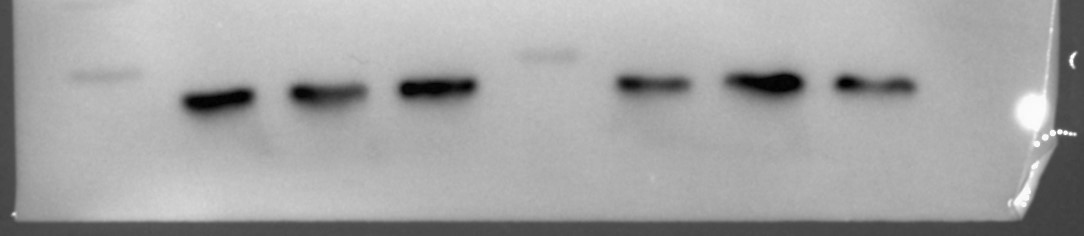


SPLA2：C11 CL11 SGB11


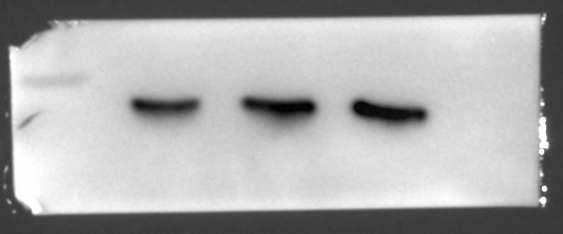

Supplement: Supplementary file 1 — Additional file 1. Western blot images. [file 12871_2022_1814_MOESM1_ESM.docx]
